# The Collection of Garbage

**Published:** 1900-08

**Authors:** 


### THE COLLECTION OF GARBAGE.

ONE can form a very fair idea of the condition of public thought, by watching closely the popular correspondence columns which every modern daily newspaper now finds it necessary to maintain for the purpose of giving free and untrammelled expression to public opinion, that invisible yet all-powerful force which shapes the destinies of men and rules the world. One matter which concerns municipal sanitation has been cropping out in Buffalo very frequently of late in these letters from the people. In this way has attention been directed to the garbage question and the fact that the collections of refuse and swill are altogether too infrequent for a city as populous and as big as Buffalo. The general trend of these letters is, that while the work is well done so far as it goes, it does not go far enough in that the garbage is taken away only twice a week. This surely, is a matter of moment and quite important enough to demand the attention of the Health Commissioner and the Board of Public Works.

As it stands now, the contractors are authorised to make collections only twice a week, and in the interval the immense and increasing supply of garbage stands in barrels and swill cans, befouling the air and making admirable garden spots for the culture of the organisms of disease. In the late fall and winter months twice a week may be sufficient for the collection of garbage; but certainly during the summer months there should be at least three general collections each week and in the more populous centers four, which would be every other day, and would not be any too frequent.

This is a matter which needs only the sanction of the Board of Public Works and a recommendation from the Health Commissioner; then there should not be any time wasted in rectifying what is palpably an oversight in a sanitary matter of the utmost moment.

